# Comparative Evaluation of Photogrammetric, Radiographic, and Direct Measurements in Facial Analysis: A Cross-Sectional Study

**DOI:** 10.7759/cureus.81445

**Published:** 2025-03-30

**Authors:** Fariha Fatima, Chandrashekhar Hallolli, Roopa Tubaki, Ieeshan Farooq Shah, Altaf H Thekiya, Humera Tabassum, Seema Gupta

**Affiliations:** 1 Department of Orthodontics and Dentofacial Orthopedics, Amrith Educational &amp; Cultural Society (AECS) Maaruti College of Dental Sciences and Research Centre, Bengaluru, IND; 2 Department of Conservative Dentistry and Endodontics, Kalka Dental College, Meerut, IND; 3 Department of Orthodontics and Dentofacial Orthopedics, Diamond Dental Care, Nanded, IND; 4 Department of Orthodontics and Dentofacial Orthopedics, Oxford Dental College, Bengaluru, IND; 5 Department of Orthodontics, Kothiwal Dental College and Research Centre, Moradabad, IND

**Keywords:** analysis, cephalometry, direct, facial, measurements, photogrammetry

## Abstract

Introduction: Facial analysis plays an important role in evaluating and planning treatments related to dental, surgical, and forensic applications. Traditionally, cephalometric radiography has been widely used to assess craniofacial structures, providing information on skeletal and dental relationships. Recently, photogrammetry has gained attention as a noninvasive method that utilizes standardized photographs to evaluate facial proportions and symmetry, eliminating radiation exposure. This study focuses on comparing photogrammetric measurements with radiographic and direct measurements to evaluate the accuracy and reliability of photogrammetry for facial analysis.

Materials and methods: This prospective, observational, cross-sectional study was conducted in the Department of Orthodontics and Dentofacial Orthopedics, Amrith Educational & Cultural Society (AECS) Maaruti College of Dental Sciences & Research Centre, Bengaluru, over two years, from September 2020 to July 2022. Fifty participants (18-25 years old) with well-balanced facial profiles were included in this study. Standardized frontal and lateral photographs were obtained under controlled lighting conditions and subject positioning. Cephalometric radiographs were obtained using digital radiography units (DRUs). Direct anthropometric measurements were recorded using a digital Vernier caliper. Eighteen cephalometric landmarks were identified for comparison purposes. Image analysis was performed using image software. Statistical analysis was conducted using Kruskal-Wallis and post hoc Dunn tests to compare the methods. Intra- and inter-examiner reliability was assessed using the intraclass correlation coefficient (ICC).

Results: The ICC values (0.85-0.91) indicated excellent measurement reliability. Photogrammetric measurements were generally higher in the horizontal frontal plane, whereas radiographic measurements yielded higher values in the vertical frontal plane. Lateral plane measurements showed greater agreement among the three methods, except for parameters such as tragon-to-subnasale distance (Trg-Sn) and alar-to-pronasale distance (Al-Prn), which differed significantly between groups.

Conclusion: Photogrammetry is a viable, noninvasive alternative to cephalometry, offering ease of use and radiation-free assessment. However, variations owing to head positioning, lack of depth perception, and minor magnification errors must be considered. Future studies should integrate three-dimensional imaging to enhance its accuracy and clinical applicability.

## Introduction

Facial analysis plays a crucial role in various fields of medicine and dentistry, including orthodontics, prosthodontics, maxillofacial surgery, and forensic science. Aesthetic and functional evaluations of the face rely on a combination of clinical examination, direct anthropometric measurements, radiographic analysis, and photogrammetric techniques [1]. Cephalometric radiography has been a cornerstone of orthodontic diagnostics, providing invaluable insights into craniofacial anatomy by capturing lateral and frontal skull images and allowing clinicians to assess skeletal and dental relationships and growth patterns [2]. The primary advantage of cephalometrics is its ability to visualize both hard and soft tissues, thereby facilitating comprehensive treatment planning. However, cephalometric radiography is not without its limitations. One significant concern is exposure to ionizing radiation, which, although minimal, raises cumulative risk considerations, especially in younger patients [3]. Additionally, landmark identification on cephalograms can be challenging because of the superimposition of bilateral structures, leading to potential inaccuracies in measurements. Moreover, cephalometric analysis predominantly focuses on two-dimensional (2D) representations of three-dimensional (3D) structures, which may not fully capture facial asymmetries or soft tissue nuances [4].

In light of these limitations, photogrammetric methods have emerged as noninvasive alternatives for facial analysis in orthodontics [5]. Photogrammetry involves obtaining precise measurements from photographs, thereby enabling the assessment of facial proportions and symmetry without radiation exposure. Standardized frontal and profile photographs are utilized in 2D photogrammetry to analyze soft tissue landmarks. This method offers advantages such as ease of acquisition, cost-effectiveness, and the ability to maintain a permanent visual record of the patient's facial features [6]. However, 2D photogrammetry is susceptible to errors arising from improper head positioning, lighting variations, and camera distortions. To mitigate these issues, rigorous standardization protocols are essential for image capture.

The accuracy of photogrammetric measurements relative to that of direct anthropometric assessments has been the subject of several studies. A study involving 30 subjects aged between 18 and 25 years compared the linear measurements obtained from standardized frontal photographs with direct facial measurements and frontal cephalograms [5]. The findings indicated that photogrammetric measurements closely approximated direct anthropometric data, with no statistically significant differences observed across the 12 parameters [5]. This suggests that when properly standardized, 2D photogrammetry can serve as a reliable tool for facial analysis in orthodontic practice [6].

Advancements in 3D photogrammetry have further enhanced the precision of facial measurement. A cross-sectional study involving 20 orthodontic patients evaluated the relationship between traditional cephalometric measurements and the corresponding non-radiographic 3D photogrammetric assessments. The study found strong positive correlations between the two methods, particularly concerning the measurements of jaw relationships and incisor orientation [7]. These results underscore the potential of 3D photogrammetry to replicate cephalometric analyses without subjecting patients to radiation, thereby aligning with the principle of "as low as reasonably achievable" (ALARA) in radiation exposure.

This study aimed to compare photogrammetric measurements with radiographic and direct facial measurements to assess the accuracy and reliability of photogrammetry for facial analysis. This study aimed to evaluate the relationship between linear measurements obtained using standardized frontal and lateral photographs, cephalometric radiographs, and direct measurements. Additionally, we sought to compare measurements derived from frontal and lateral photographic and radiographic analyses to determine their consistency and applicability in clinical diagnostics. Furthermore, this study aimed to assess the reliability of photogrammetry as a diagnostic tool in orthodontics, exploring its potential as a noninvasive alternative to traditional cephalometric and direct measurement methods.

## Materials and methods

Study design, setting, and sample size

This prospective, observational, cross-sectional study was conducted in the Department of Orthodontics and Dentofacial Orthopedics at Amrith Educational & Cultural Society (AECS) Maaruti College of Dental Sciences & Research Centre, Bengaluru over a span of two years from September 2020 to July 2022. This study adhered to the principles of the Declaration of Helsinki and obtained prior approval from the institutional ethical committee of the College (AECS/MDC/151/2019-20, dated October 21, 2019). Written informed consent was obtained from all the patients. Sample size estimation was conducted using G*Power software version 3.2.9 (Heinrich-Heine-Universität Düsseldorf, Düsseldorf, Germany) to achieve a statistical power of 80% with a significance level (alpha error) of 5%. Based on a minimum effect size of 0.17, derived from a prior study by Jaiswal et al., a total sample size of 50 was sufficient for robust statistical analysis [8].

Eligibility criteria

The sample consisted of 50 Indian participants, both male and female, aged between 18 and 25 years, selected from undergraduate and postgraduate students as well as patients at the dental college. All participants exhibited a well-balanced face and pleasing soft tissue profile. Standard cephalometric radiographs and both frontal and lateral photographs were used as data sources. Before obtaining cephalograms and photographs, participants’ demographic details, such as name, age, and sex, were recorded. The inclusion criteria were as follows: subjects belonging to the Indian population within the specified age group, exhibited a bilateral class I molar relationship, had minimal dental crowding or spacing (< 2 mm), had all permanent teeth erupted up to the second molar, and presented a well-balanced facial profile. Individuals with a history of orthodontic treatment, orthognathic surgery, craniofacial trauma, temporomandibular joint disorders, periodontal disease, or congenital abnormalities were excluded from the study.

Methodology

For the study, 19 common cephalometric landmarks were identified and marked on both lateral and frontal radiographs and photographs of 50 participants, chosen based on ease of identification and reproducibility (Table 1).

**Table 1:** Anatomical landmarks used in the study with description.

S No.	Landmarks	Description
1.	Trichion (Tri)	The sagittal midpoint of the forehead that borders the hairline.
2.	Glabella (G)	The most prominent or anterior point in the midsagittal plane of the forehead at the level of superior orbital ridges.
3.	Nasion (N)	It is the concave or retruded point in the tissue overlying the area of the frontonasal suture.
4.	Orbitale (O)	It is the lowest point on the lower margin of each orbit. It is identified by palpation and is identical to the bony orbitale.
5.	Zygoma (Zyg)	Zygoma is the centre of zygomatic arch by inspection for frontal.
6.	Pronasale (Prn)	It is the most prominent or anterior point of the nose.
7.	Antegonion (Ag)	It is the highest point in the antegonial notch.
8.	Pogonion (Pog)	The most prominent point on the chin.
9.	Gnathion (Gn)	The deepest point on the chin.
10.	Menton (Me)	The most inferior midline point on the mandibular symphysis.
11.	Endocanthus (En)	It is the medial canthus of the eye.
12.	Exocanthus (Ex)	It is the lateral canthus of the eye.
13.	Subnasale (Sn)	Midpoint of the columella base at the apex of the nasolabial angle.
14.	Labrale superius (Ls)	A point indicating the mucocutaneous border of the upper lip.
15.	Labrale inferius (Li)	A point indicating the mucocutaneous border of the lower lip.
16.	Stomium superius (Sts)	The lowermost point on the vermilion of the upper lip.
17.	Stomium inferius (Sti)	The uppermost point on the vermilion of the lower lip.
18.	Tragus (Trg)	The most prominent part of the articular tragus.
19.	Alar (Al)	The most lateral point of the alar contour of the nose.

**Figure 1:**
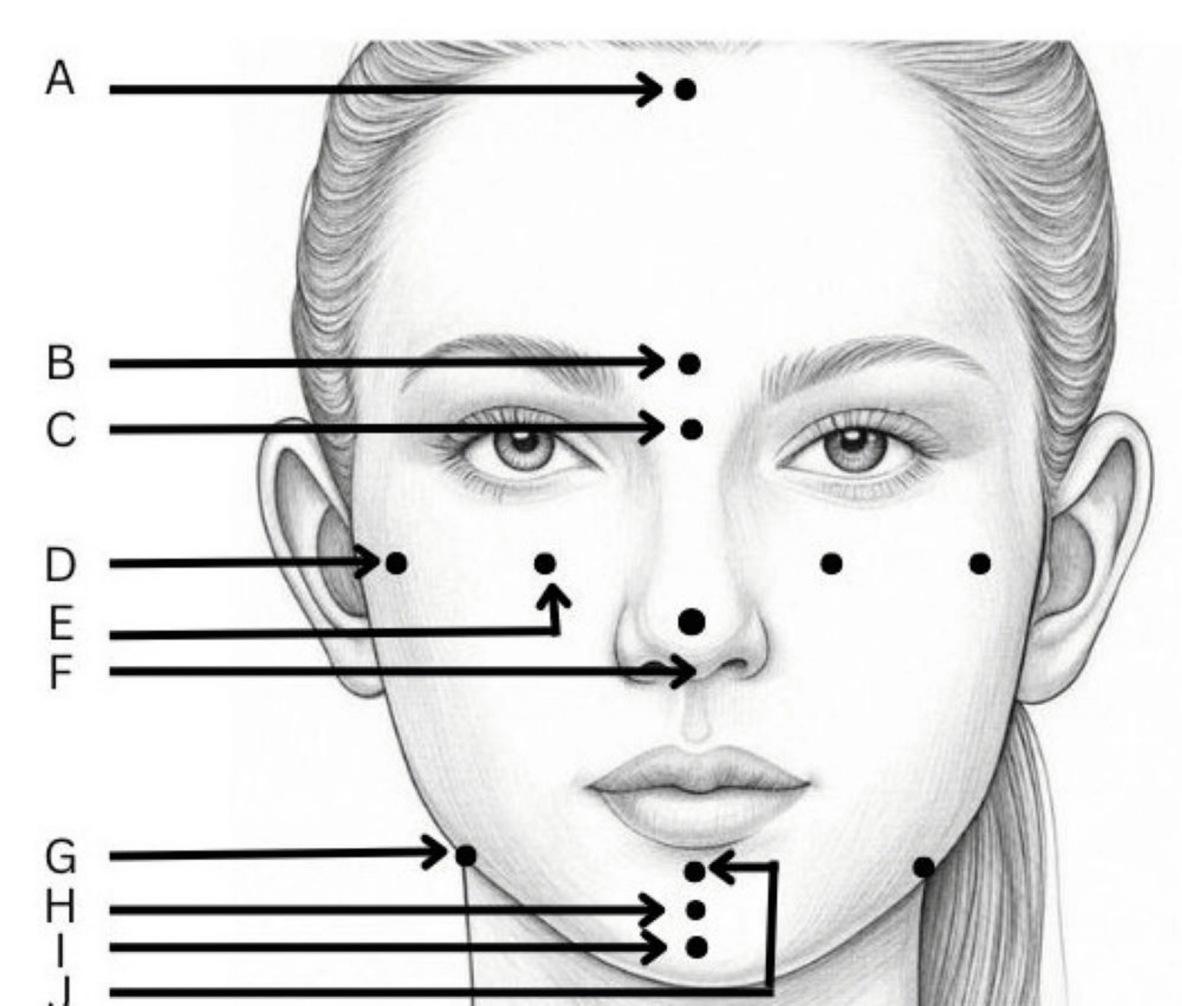
Anatomical landmarks used in the study for frontal plane analysis. A: Trichion (Tri), B: Glabella (G), C: Nasion (N), D: Orbitale (O), E: Zygoma (Zyg), F: Pronasale (Prn), G: Antegonion (Ag), H: Pogonion (Pog), I: Gnathion (Gn), J: Menton (Me). Author created the schematic diagram of a female face.

Standardized photographs were taken using a Canon EOS 1200D (DSLR) camera equipped with a Canon EF-S 18-55 mm F/3.5-5.6 zoom lens (Canon Inc., Tokyo, Japan). The camera was mounted on a height-adjustable tripod, positioned three feet from the subject, and set to manual mode with a shutter speed of 1/125/s and an aperture of f/11 to ensure consistency in exposure. Photographs were recorded at a 1:1 ratio to obtain life-size measurements. Two Simpex Pro 150 W studio lights (Simpex, Maharashtra, India) were used for the uniform lighting. Frontal and lateral photographs were captured with the subject in a natural head position (NHP) and with the lips at rest. To achieve NHP, subjects were seated upright on a height-adjustable chair and asked to look into a mirror placed at eye level. A modified protractor with a plumb bob was used to confirm the NHP in both the frontal and profile views. A metal scale was suspended parallel to the midsagittal plane for true vertical reference and photographic calibrations. Stainless steel markers were placed on anatomical landmarks to minimize landmark identification errors (Figure 1).

Frontal and lateral cephalometric radiographs were obtained using an Avanttec 8000 C (Avanttec Medical Systems, Carestream Health, NY, USA) digital radiography unit. The exposure parameters were standardized to 78 kV, 10 mA, and 0.50 seconds. Radiographs were taken in the NHP and confirmed using the cephalostat’s laser alignment along the Porion-Orbitale (Frankfort Horizontal) plane. A pre-set scale measurement of the cephalostat ensured radiographic calibration. Direct facial measurements were obtained with a digital Vernier caliper in the NHP with the subject’s lips relaxed. Thirteen linear measurements were recorded in the frontal view and 15 in the lateral view (Tables 2, 3).

**Table 2:** Measurements in frontal plane used in the study. En: endocanthus, Exr: exocanthus right, Exl: exocanthus left, Or: orbitale right, Ol: orbitale left, Zygr: zygoma right, Zygl: zygoma left, Alr: alar right, All: alar left, Agr: antegonion right, Agl: antegonion left, N: nasion, Sn: subnasale, Me: menton, Pog: pogonion, Gn: gnathion. All the measurements are in mm.

S No.	Measurements
1.	En right (r)- En left (l)
2.	Exr-Exl
3.	Biorbitale width from Or-Ol
4.	Bizygomatic width from Zygr-Zygl
5.	Alar base width from Alr-All
6.	Antegonial width from Agr-Agl
7.	Midfacial length from N-Sn
8.	Lower facial length from Sn-Me
9.	Anterior facial length from N-Me
10.	N-Pog
11.	Sn-Pog
12.	Pog-Me
13.	Sn-Gn

**Table 3:** Measurements in lateral plane used in the study. Trg: tragus, Sn: subnasale, Al: alar, Prn: pronasale, N: nasion, O: orbitale, Sn: subnasale, Ls: labrale superius, Li: labrale inferius, Pog: pogonion, G: glabella, Me: menton, Sts: stomium superius, Sti: stomium inferius. All the measurements are in mm.

S No.	Measurements
1.	Facial depth from Trg-Sn
2.	Nasal depth from Al-Prn
3.	Nasal prominence from Prn-NO line
4.	Subnasale depth from Sn-NO line
5.	Upper lip prominence from Ls-NO line
6.	Lower lip prominence from Li-NO line
7.	Chin prominence from Pog-NO line
8.	Upper facial third from Tri-G
9.	Mid facial third from G-Sn
10.	Lower facial third from Sn-Me
11.	Nasal length from N-Sn
12.	Upper lip length from Sn-Sts
13.	Lower lip length from Sti-Me
14.	Interlabial gap from Sts-Sti
15.	Height of the nasal tip from Sn-Prn

The collected photographs and radiographs were digitized and analyzed using the VistaDent OC Software (Dentsply Sirona, North Carolina, US). Image calibration was performed using the software, with photographs set to a default 1:1 magnification ratio, while radiographs were calibrated to 92% (0.092 magnification) due to inherent magnification differences. The software allowed for precise landmark identification and measurement. Measurements from frontal and lateral photographs and radiographs were systematically recorded and used for statistical analysis to evaluate and compare between the photogrammetric, radiographic, and direct anthropometric measurements. All measurements were conducted by two calibrated examiners, who repeated all measurements on randomly selected patients after one month. Inter- and intra-examiner reliability were assessed using the intraclass correlation coefficient (ICC) test.

Statistical analysis

Statistical analysis was performed using IBM SPSS Statistics for Windows, version 23 (IBM Corp., Armonk, NY). The normality of data was assessed using the Shapiro-Wilk test, and non-normal distribution was confirmed by a histogram/Q-Q plot. Continuous data were presented as mean and standard deviation. Three methodological groups were compared using the Kruskal-Wallis test (nonparametric), followed by the post hoc Dunn test. Statistical significance was set at p < 0.05 as a significant level.

## Results

The intra- and inter-examiner reliabilities were in the range of 0.85-0.91, which showed excellent reliability and reproducibility. The comparison of frontal measurements in the horizontal plane using the Kruskal-Wallis test revealed significant differences among the three methods for most variables. The mean Enr-Enl measurement was highest for the photogrammetric method and lowest for the radiographic method (p = 0.001). Similarly, for Exr-Exl, the radiographic method showed the highest mean value, whereas the photogrammetric method showed the lowest (p = 0.001). No significant differences were observed in the Or-Ol across the three methods (p = 0.515). The Zygr-Zygl measurements varied significantly, with the highest mean recorded in the direct method and the lowest in the radiographic method (p = 0.001). Agr-Agl measurements were significantly different, with the photogrammetric method showing the highest mean and the direct method showing the lowest value (p = 0.001). Finally, Alr-All also exhibited a significant difference, with the photogrammetric method yielding the highest value and the radiographic method yielding the lowest value. These findings indicate that the measurement methods influence the obtained values, with photogrammetric and direct methods often yielding larger measurements than radiographic methods (Table 4).

**Table 4:** Comparison of frontal measurements in horizontal plane by Kruskal-Wallis test. *p-value < 0.05: significant, data is presented in the form of mean and standard deviation (SD). Enr: endocanthus right, Enl: endocanthus left, Exr: exocanthus right, Exl: exocanthus left, Or: orbitale right, Ol: orbitale left, Zygr: zygoma right, Zygl: zygoma left, Agr: antegonion right, Agl: antegonion left, Alr: alar right, All: alar left. All the measurements are in mm.

Variables	Group	Frequency	Mean	SD	Statistics	p-value
Enr-Enl	Photogrammetric	50	33.12	2.15	61.87	0.001*
	Radiographic	50	29.28	0.76		
	Direct	50	30.29	2.23		
Exr-Exl	Photogrammetric	50	97.30	2.05	46.77	0.001*
	Radiographic	50	100.39	3.39		
	Direct	50	99.17	3.96		
Or-Ol	Photogrammetric	50	51.76	6.82	1.33	0.515
	Radiographic	50	50.81	4.29		
	Direct	50	50.93	3.89		
Zygr-Zygl	Photogrammetric	50	120.37	1.49	24.16	0.001*
	Radiographic	50	119.91	3.08		
	Direct	50	122.14	2.86		
Agr-Agl	Photogrammetric	50	96.82	2.59	36.8	0.001*
	Radiographic	50	93.88	4.30		
	Direct	50	93.61	4.79		
Alr-All	Photogrammetric	50	36.42	1.81	26.48	0.001*
	Radiographic	50	32.76	3.56		
	Direct	50	34.46	2.00		

Post hoc analysis for pairwise group comparisons of frontal measurements in the horizontal plane revealed significant differences between methods. The Enr-Enl and Exr-Exl measurements showed significant differences between the photogrammetric and radiographic methods as well as between the photogrammetric and direct methods. Zygr-Zygl, Agr-Agl, and Alr-All measurements also demonstrated significant differences between the photogrammetric and direct methods. Additionally, the Exr-Exl and Zygr-Zygl measurements differed significantly between radiographic and direct methods. These results suggest that the horizontal plane measurements vary across different methods, with notable discrepancies in the Enr-Enl, Exr-Exl, and Zygr-Zygl dimensions (Table 5).

**Table 5:** Post hoc analysis for pairwise group comparison of frontal measurements in horizontal plane by Dunn test. *p-value < 0.05: significant, all the values in the table are the p-values. Enr: endocanthus right, Enl: endocanthus left, Exr: exocanthus right, Exl: exocanthus left, Or: orbitale right, Ol: orbitale left, Zygr: zygoma right, Zygl: zygoma left, Agr: antegonion right, Agl: antegonion left, Alr: alar right, All: alar left.

Intergroup comparison	Enr-Enl	Exr-Exl	Or-Ol	Zygr-Zygl	Agr-Agl	Alr-All
Photogrammetric - Radiographic	0.001*	0.001*	0.259	0.206	0.001*	0.001*
Photogrammetric - Direct	0.001*	0.001*	0.44	0.001*	0.001*	0.001*
Radiographic - Direct	0.079	0.017*	0.722	0.001*	0.732	0.673

The comparison of frontal measurements in the vertical plane using the Kruskal-Wallis test showed significant differences among the three methods for most variables. The mean N-Sn concentration was the highest in the photogrammetric method and the lowest in the direct method (p = 0.001). Sn-Me showed significantly higher values with the radiographic method than with the photogrammetric method, which yielded the lowest value. For N-Me, the radiographic method recorded the highest mean value, whereas the photogrammetric method recorded the lowest value. The N-Pog measurement yielded the highest mean value, whereas the direct method yielded the lowest value. Sn-Pog showed the highest mean value with the photogrammetric method, whereas the direct method gave the lowest value. Similarly, Pog-Me differed significantly, with the photogrammetric method yielding the highest mean value and the direct method yielding the lowest value. Finally, the radiographic method yielded the highest mean value for Sn-Gn, and the photogrammetric method yielded the lowest value. These findings suggest that radiographic methods often yield larger vertical measurements than the photogrammetric and direct methods (Table 6).

**Table 6:** Comparison of frontal measurements in vertical plane by Kruskal-Wallis test. *p-value < 0.05: significant, data is presented in the form of mean and standard deviation (SD). N: nasion, Sn: subnasale, Me: menton, Pog: pogonion, Gn: gnathion. All the measurements are in mm.

Variables	Group	Frequency	Mean	SD	Statistics	p-value
N-Sn	Photogrammetric	50	56.62	3.18	43.28	0.001*
	Radiographic	50	54.90	2.60		
	Direct	50	53.20	2.59		
Sn-Me	Photogrammetric	50	62.20	3.07	6.37	0.041*
	Radiographic	50	63.24	3.02		
	Direct	50	63.17	3.90		
N-Me	Photogrammetric	50	99.33	4.78	13.58	0.001*
	Radiographic	50	101.47	5.64		
	Direct	50	99.60	4.10		
N-Pog	Photogrammetric	50	94.94	3.44	7.95	0.019*
	Radiographic	50	95.19	4.42		
	Direct	50	93.82	4.33		
Sn-Pog	Photogrammetric	50	57.71	3.81	16.38	0.001*
	Radiographic	50	56.32	2.48		
	Direct	50	56.02	2.47		
Pog-Me	Photogrammetric	50	10.54	0.71	19.07	0.001*
	Radiographic	50	10.20	1.62		
	Direct	50	9.85	1.76		
Sn-Gn	Photogrammetric	50	54.70	2.74	9.20	0.01*
	Radiographic	50	56.22	2.15		
	Direct	50	55.89	3.25		

Post hoc analysis for pairwise group comparisons of frontal measurements in the vertical plane revealed several significant differences. The N-Sn, Sn-Me, N-Me, Pog-Me, and Sn-Gn measurements showed significant differences between photogrammetric and radiographic methods. Similarly, the N-Sn, Sn-Me, N-Pog, Sn-Pog, and Pog-Me measurements differed significantly between the photogrammetric and direct methods. Additionally, the N-Sn, N-Me, and N-Pog measurements showed significant differences between radiographic and direct methods. These findings indicate that frontal measurements in the vertical plane exhibit variability across different methods, with notable discrepancies observed in N-Sn, N-Me, and Pog-Me measurements (Table 7).

**Table 7:** Post hoc analysis for pairwise group comparison of frontal measurements in vertical plane by Dunn test. *p-value < 0.05: significant, all the values in the table are the p-values. N: nasion, Sn: subnasale, Me: menton, Pog: pogonion, Gn: gnathion.

Intergroup comparison	N-Sn	Sn-Me	N-Me	N-Pog	Sn-Pog	Pog-Me	Sn-Gn
Photogrammetric - Radiographic	0.003*	0.029*	0.001*	0.940	0.001*	0.006*	0.003*
Photogrammetric - Direct	0.001*	0.029*	0.087	0.013*	0.001*	0.001*	0.078
Radiographic - Direct	0.001*	0.996	0.049*	0.016*	0.611	0.116	0.208

A comparison of the lateral measurements in the horizontal plane using the Kruskal-Wallis test revealed no statistically significant differences among the three methods for any of the variables. No significant differences were observed between the methods (p > 0.05). These results suggest that different measurement techniques do not substantially impact the lateral measurements in the horizontal plane, and any observed variations are likely to be within the range of normal measurement discrepancies (Table 8).

**Table 8:** Comparison of lateral measurements in horizontal plane by Kruskal-Wallis test. *p-value > 0.05: non-significant, data is presented in the form of mean and standard deviation (SD). Trg: tragus, Sn: subnasale, N: nasion, O: orbitale, Al: alar, Prn: pronasale, Ls: labrale superius, Li: labrale inferius, Pog: pogonion. All the measurements are in mm.

Variables	Group	Frequency	Mean	SD	Statistics	p-value
Trg-Sn	Photogrammetric	50	97.96	5.39	4.20	0.122
	Radiographic	50	100.24	5.87		
	Direct	50	97.39	8.66		
Al-Prn	Photogrammetric	50	26.74	2.82	5.10	0.078
	Radiographic	50	26.21	2.03		
	Direct	50	27.41	2.23		
Prn-NO line	Photogrammetric	50	18.27	1.80	3.76	0.152
	Radiographic	50	18.45	2.42		
	Direct	50	18.74	3.52		
Sn-NO line	Photogrammetric	50	5.51	3.81	3.36	0.187
	Radiographic	50	6.30	3.88		
	Direct	50	6.16	3.97		
Ls-NO line	Photogrammetric	50	6.54	4.93	0.16	0.921
	Radiographic	50	6.96	5.24		
	Direct	50	6.58	5.31		
Li-NO line	Photogrammetric	50	6.52	5.81	0.27	0.873
	Radiographic	50	6.68	5.88		
	Direct	50	6.97	5.96		
Pog-NO line	Photogrammetric	50	8.91	7.97	0.37	0.831
	Radiographic	50	9.24	8.20		
	Direct	50	8.83	8.38		

Post hoc analysis for pairwise group comparisons of lateral measurements in the horizontal plane revealed significant differences between the two parameters. The Trg-Sn measurement showed a significant difference between the photogrammetric and radiographic methods, whereas the Al-Prn measurement differed significantly between the radiographic and direct methods. No other comparisons showed statistically significant differences between the groups. These findings suggest that Trg-Sn measurements vary more between photogrammetric and radiographic methods, whereas Al-Prn measurements show discrepancies between radiographic and direct methods. However, overall, most lateral measurements in the horizontal plane were consistent across the three methods (Table 9).

**Table 9:** Post hoc analysis for pairwise group comparison of lateral measurements in horizontal plane by Dunn test. *p-value < 0.05: significant, all the values in the table are the p-values. Trg: tragus, Sn: subnasale, N: nasion, O: orbitale, Al: alar, Prn: pronasale, Ls: labrale superius, Li: labrale inferius, Pog: pogonion.

Intergroup comparison	Trg-Sn	Al-Prn	Prn-NO line	Sn-NO line	Ls-NO line	Li-NO line	Pog-NO line
Photogrammetric - Radiographic	0.041*	0.686	0.394	0.073	0.681	0.833	0.725
Photogrammetric - Direct	0.490	0.085	0.054	0.223	0.801	0.604	0.799
Radiographic - Direct	0.185	0.034*	0.279	0.566	0.882	0.758	0.545

A comparison of lateral measurements in the vertical plane using the Kruskal-Wallis test showed significant differences among the three methods for several variables. The mean Tri-G measurement was highest in the radiographic method and lowest in the photogrammetric method (p = 0.023). Similarly, G-Sn was highest in the radiographic method and lowest in the photogrammetric method (p = 0.034). The N-Sn measurement also showed the highest mean value using the radiographic method and the lowest mean value using the photogrammetric method. Sts-Sti demonstrated the highest value in the radiographic method, whereas the photogrammetric method gave the lowest value. Sti-Me also showed the highest value with the radiographic method, whereas the photogrammetric method gave the lowest value. However, Sn-Me, Sn-Sts, and Sn-Prn did not exhibit statistically significant differences between the three measurement methods. These findings suggest that radiographic measurements tend to yield higher values than photogrammetric and direct methods for certain vertical-plane measurements, which may be attributed to differences in imaging perspectives and magnification effects (Table 10).

**Table 10:** Comparison of lateral measurements in vertical plane by Kruskal-Wallis test. *p-value < 0.05: significant, data is presented in the form of mean and standard deviation (SD). Tri: trichion, G: glabella, Sn: subnasale, Me: menton, N: nasion, Prn: pronasale, Sts: stomium superius, Sti: stomium inferius. All the measurements are in mm.

Variables	Group	Frequency	Mean	SD	Chi-value	p-value
Tri-G	Photogrammetric	50	42.58	6.88	7.51	0.023*
	Radiographic	50	44.54	6.39		
	Direct	50	43.95	6.94		
G-Sn	Photogrammetric	50	58.64	3.88	6.75	0.034*
	Radiographic	50	59.86	4.29		
	Direct	50	59.62	3.70		
Sn-Me	Photogrammetric	50	61.72	6.27	4.33	0.115
	Radiographic	50	63.42	5.24		
	Direct	50	62.44	4.40		
N-Sn	Photogrammetric	50	48.42	3.74	10.18	0.006*
	Radiographic	50	50.11	3.21		
	Direct	50	49.45	3.61		
Sn-Sts	Photogrammetric	50	16.61	2.50	5.10	0.078
	Radiographic	50	17.15	3.09		
	Direct	50	17.63	2.73		
Sts-Sti	Photogrammetric	50	0.61	0.40	8.84	0.012*
	Radiographic	50	0.88	0.50		
	Direct	50	0.75	0.29		
Sti-Me	Photogrammetric	50	44.53	3.02	12.80	0.002*
	Radiographic	50	46.63	3.21		
	Direct	50	45.47	3.71		
Sn-Prn	Photogrammetric	50	11.89	3.21	0.88	0.644
	Radiographic	50	11.97	3.29		
	Direct	50	12.00	2.40		

Post hoc analysis for pairwise group comparisons of lateral measurements in the vertical plane revealed several significant differences among the three measurement methods. The Tri-G and G-Sn measurements showed significant differences between the three methods. Sn-Me differed significantly between the photogrammetric and radiographic methods. The N-Sn measurement was significantly different between the photogrammetric and radiographic methods and between the photogrammetric and direct methods. Additionally, Sn-Sts showed a significant difference between photogrammetric and direct methods. Sts-Sti differed significantly between the photogrammetric and radiographic methods. Sti-Me showed a significant difference between the photogrammetric and radiographic methods. No significant differences were observed between the radiographic and direct methods for any of the parameters. These results indicate that photogrammetric measurements tend to differ more significantly from radiographic and direct methods, whereas radiographic and direct methods showed greater agreement in vertical plane measurements (Table 11).

**Table 11:** Post hoc analysis for pairwise group comparison of lateral measurements in vertical plane by Dunn test. *p-value < 0.05: significant, all the values in the table are the p-values. Tri: trichion, G: glabella, Sn: subnasale, Me: menton, N: nasion, Prn: pronasale, Sts: stomium superius, Sti: stomium inferius.

Intergroup comparison	Tri-G	G-Sn	Sn-Me	N-Sn	Sn-Sts	Sts-Sti	Sti-Me	Sn-Prn
Photogrammetric - Radiographic	0.009*	0.037*	0.038*	0.002*	0.431	0.004*	0.001*	0.744
Photogrammetric - Direct	0.043*	0.017*	0.358	0.029*	0.026*	0.051	0.059	0.355
Radiographic - Direct	0.561	0.765	0.265	0.352	0.151	0.336	0.091	0.549

## Discussion

This study aimed to compare and correlate photogrammetric measurements with radiographic and direct facial measurements to assess the accuracy and reliability of photogrammetry for facial analysis. These findings highlight both the advantages and limitations of photogrammetric techniques compared with traditional cephalometric and direct anthropometric measurements.

The results indicated significant differences between the photogrammetric, radiographic, and direct measurement methods in the frontal and lateral dimensions. Photogrammetric measurements tend to be larger in the horizontal frontal plane, whereas radiographic methods often yield higher values in the vertical frontal plane. This might be due to the fact that photographs capture external soft tissue contours, which may appear wider in the frontal plane due to skin and muscle tension, while the radiographs penetrate soft tissues and primarily measure skeletal structures, leading to higher vertical values where the bone structure is dominant. In photogrammetric analysis, even slight tilting or rotation of the head can affect the frontal horizontal dimensions, making them appear broader than actual. On radiographs, the patient is often positioned in a standardized head posture, reducing errors in the vertical dimension assessment. The tendency of photogrammetric methods to overestimate transverse facial dimensions aligns with the findings of Hajeer et al., who reported that improper head positioning and camera distortions can result in larger-than-actual measurements [9]. Conversely, radiographic measurements exhibited higher vertical values, a finding consistent with the study by Song et al., which suggested that cephalometric radiographs tend to magnify vertical dimensions because of patient positioning and inherent radiographic distortion [10]. The highest values in the Sn-Me and N-Me distances recorded on radiographs may be a consequence of cephalometric magnification and differences in landmark identification criteria.

The radiographic method in our study showed a non-significant difference from the direct method for most frontal measurements in both the horizontal and vertical planes, compared to the photogrammetric method. These discrepancies may be attributed to variations in soft tissue thickness among individuals, which affected the measurements performed using photographs. Similar results were reported by Benson and Richmond, who reported that the photogrammetric method is not a valid and reproducible method for soft tissue measurements compared to radiographs, owing to the distortion factor [11]. According to Grybauskas et al., although significant differences were observed between radiographic and photogrammetric measurements, these differences were clinically insignificant and smaller than one unit of measurement [12].

The significant differences observed in the frontal plane measurements between the photogrammetric and radiographic methods highlight the necessity of calibration and standardization in image acquisition. Post hoc analysis further confirmed that photogrammetric and radiographic methods diverge most significantly in horizontal plane dimensions, suggesting that while photogrammetry is valuable, it cannot entirely replace radiographic methods in certain cases, particularly when precise skeletal assessments are needed.

On the other hand, the findings of this study indicated that lateral plane measurements exhibited greater agreement among the three methods, except for a few parameters such as Trg-Sn and Al-Prn. This suggests that lateral measurements are less affected by methodological variations, potentially rendering photogrammetry a reliable tool for lateral profile assessments. These results are in line with those of de Carvalho Rosas Gomes et al. and Farkas et al., who showed that lateral photogrammetric measurements were more comparable to cephalometric values than to frontal measurements [13,14]. Farkas et al. also concluded that most of the photogrammetric measurements varied by no more than 1 mm when compared with direct measurements [14]. Zhang et al. undertook an investigation to evaluate craniofacial metrics derived from cephalometric radiographs in relation to the corresponding metrics obtained from standardized facial photographs of a cohort of 326 subjects [15]. The precision of the photogrammetric methodology exhibited a high degree of reliability, as evidenced by all the measurements, yielding an ICC > 0.90. Conversely, the associations between the corresponding photogrammetric and cephalometric measurements were lower, demonstrating variability within the range 0.356-0.643. The most pronounced correlations were identified for the lower facial height and mandibular length (0.643 and 0.562, respectively).

Mehta et al. conducted a comparative study in skeletal class II patients, where craniofacial lateral measurements were compared between radiographic and photogrammetric methods, and it was found that most lateral measurements showed a good relationship between both methods; therefore, they recommended the use of the photogrammetric method reliably and reproducibly following a standardized protocol for facial measurements [16]. Similar results were reported by Gupta et al. [17]. A good correlation was found between both methods for four linear and seven angular measurements. However, none of these studies evaluated parameters in both the frontal and vertical planes and compared them with direct measurements, as in the present study. Negi et al. compared radiographic and photogrammetric measurements with direct measurements in the frontal plane only and found that among all the frontal measurements, Or-Ol showed a high correlation [5]. There was a moderate correlation with Enr-Enl, and a highly significant correlation was evident between N-Sn and Agr-Agl.

Clinical implications of the study

Photogrammetric techniques offer several advantages, including a noninvasive nature, cost-effectiveness, and elimination of radiation exposure. These benefits make photogrammetry particularly attractive for longitudinal growth studies and routine orthodontic assessments, particularly in younger patients where cumulative radiation exposure is a concern. The principle of ALARA further supports the transition towards non-radiographic methods where feasible. However, cephalometric analysis continues to be the preferred methodology for patient management in clinical settings, and photographic documentation may prove advantageous for extensive epidemiological investigations across various clinical and field environments using a standardized protocol.

Limitations of the study and future recommendations

This study has several limitations that should be acknowledged. First, the sample consisted exclusively of Indian participants aged 18-25 years, which limits the generalizability of the findings to other age groups and ethnic populations. Facial anthropometric measurements can vary significantly across ethnicities, and the absence of a diverse sample restricts the applicability of the results. Second, sex-based differences in facial measurements were not evaluated. Males and females exhibit distinct craniofacial characteristics, and not analyzing these variations may have influenced the accuracy of comparisons between measurement methods. Third, photogrammetric analysis is highly dependent on image-acquisition protocols. Although efforts have been made to standardize head positioning and lighting, minor variations in posture, camera angle, and soft-tissue tension may have introduced measurement inconsistencies. Fourth, this study relied on 2D photogrammetry, which does not fully capture the depth and 3D structure of the face. A 3D photogrammetric approach can provide more accurate and comprehensive soft tissue assessment. Finally, cephalometric radiographs inherently involve magnification errors, which may have influenced the comparison between the direct and photogrammetric measurements. Future studies should incorporate additional validation techniques, such as cone-beam computed tomography (CBCT) or 3D stereophotogrammetry, to enhance accuracy.

## Conclusions

The results demonstrated that although photogrammetry is a practical and noninvasive method, it exhibits variations in accuracy depending on the measurement plane and specific facial landmarks. Photogrammetric measurements tended to be larger in the horizontal frontal plane, whereas radiographic methods often yielded higher values in the vertical frontal plane. However, the lateral plane measurements exhibited greater agreement among the three methods, suggesting that lateral plane dimensions can be reliably captured using photogrammetry. Photogrammetry offers advantages such as ease of use, non-invasiveness, and elimination of radiation exposure, making it a valuable tool in clinical and research settings. However, inherent limitations, including variations due to head positioning, lack of depth perception, and minor magnification errors, must be considered when interpreting the results. Future studies should incorporate 3D imaging to enhance accuracy and address these limitations.
